# Protective Effects of Wild *Sulla coronaria* (Fabaceae) Flowers Phytocomplex in Human Dermal Fibroblasts Stimulated with Interleukin-1β

**DOI:** 10.3390/plants13192748

**Published:** 2024-09-30

**Authors:** Giuseppe Antonio Malfa, Simone Bianchi, Vivienne Spadaro, Ekaterina Kozuharova, Pasquale Marino, Francesco Pappalardo, Claudia Di Giacomo, Rosaria Acquaviva

**Affiliations:** 1 Department of Drug and Health Sciences, University of Catania, Viale A. Doria 6, 95125 Catania, Italy; simone.bianchi@phd.unict.it (S.B.); francesco.pap82@gmail.com (F.P.); cdigiaco@unict.it (C.D.G.); racquavi@unict.it (R.A.); 2 Research Centre on Nutraceuticals and Health Products (CERNUT), University of Catania, Viale A. Doria 6, 95125 Catania, Italy; 3 PLANTA/Center for Research, Documentation and Training, Via Serraglio Vecchio 28, 90123 Palermo, Italy; 4 Department of Biological, Chemical and Pharmaceutical Sciences and Technologies, Section of Botany, Anthropology and Zoology, University of Palermo, Via Archirafi 38, 90123 Palermo, Italy; vivienne.spadaro@unipa.it; 5 Department of Pharmacognosy, Faculty of Pharmacy, Medical University-Sofia, 1000 Sofia, Bulgaria; ina_kozuharova@yahoo.co.uk

**Keywords:** *Hedysarum coronarium*, Sicilian vascular flora, polyphenols, flavonols, quercetin, ROS, nitrites/nitrates, NO•

## Abstract

*Sulla coronaria* is indigenous to the Mediterranean region. It is grown as fodder in southern Italy because it contains various secondary metabolites with beneficial activities on animals. Recently, its potential use in cosmeceutical treatments for skin problems was reported. In this scenario, to contribute to a possible cosmeceutical application, we characterized the phytochemical profile of *Sulla coronaria* flowers’ hydroalcoholic extract by HPLC-DAD, Folin-Ciocalteu, Aluminum Chloride methods, DPPH assay, and, for the first time, we evaluated the antioxidant and anti-inflammatory activities on dermal fibroblasts. The phytochemical analysis confirmed the significant content of phenolic compounds (TPC 69.8 ± 0.6 mg GAE/g extract, TFC 15.07 mg CE/g extract) and the remarkable presence of rutin, quercetin, and isorhamnetin derivatives that give to the phytocomplex a good antioxidant activity as highlighted by the DPPH assay (IC_50_ of 8.04 ± 0.5 µg/mL). Through the reduction in NO• and ROS levels in human dermal fibroblasts, the biological tests demonstrated both the safety of the extract and its ability to counteract the inflammatory state generated by Interleukin-1β exposure. Our findings indicate that the antioxidant activities of the phytocomplex are strictly related to the anti-inflammatory action of the *Sulla coronaria* flowers extract, confirming that this plant could be a valuable source of bioactive molecules for cosmeceutical and nutraceutical applications.

## 1. Introduction

**Figure 1:**
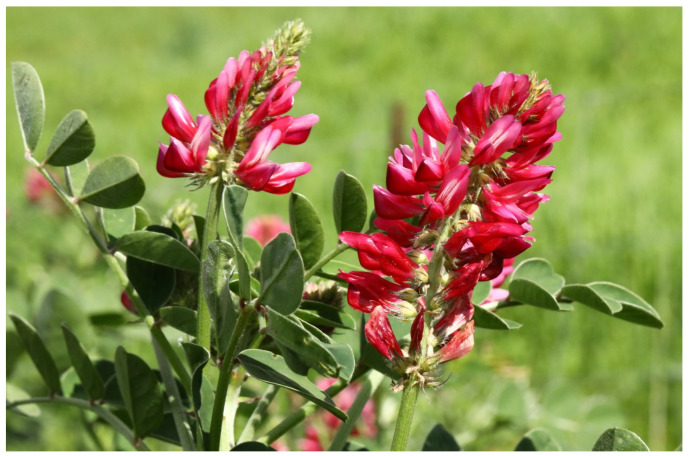
Blooming of wild *Sulla coronaria* plant at the collection site (Corleone, Palermo, Italy).

In the genus *Sulla* Medik. (Fabaceae), seven species are present differently distributed in the Mediterranean region [1]. Among them, *Sulla coronaria* (L.) Medik. ex B.H.Choi and H.Ohashi [syn. *Hedysarum coronarium* L.] is the only perennial species in the genus widely farmed for ecological and agricultural benefits [1]. Agriculture greatly benefits from the plant’s capacity to generate high-quality rich in protein feed and its capacity to fix nitrogen from the atmosphere in soil, removing the need for nitrogen fertilizers [2]. Its drought tolerance and capacity to grow in various soil types further contribute to its viability for cultivation in a wide range of climates [2]. In addition, its extensive root system helps to stabilize the soil by stopping erosion and promoting soil preservation. The flowers of this plant, gathered in ovoid axillary racemes, are pedunculated, with a ruby-red to violet corolla with a 15–20 mm vexillum and calyx 7–8 mm with subequal teeth (Figure 1) [3]. From these flowers, bees make Sulla honey, a distinct monofloral nectar produced in south Italy and Sicily with demonstrated health-beneficial properties due to high polyphenol content [4]. Traditionally, in Sicily, flowering tops are eaten in mixed salads or used to prepare omelets and soups [5]. In herbal medicine, its dried fragrant flowers are used for their astringent properties and cholesterol-lowering and laxative preparations [6]. To date, scientific studies on its potential health benefits are scarce and are sustained only by the significant amount of secondary metabolites and by its good nutritional properties [7,8,9]. Only a few studies are present in the scientific literature on the chemical composition of this plant, and only one concerns the composition of the flowers, which reported the presence of numerous flavonoids such as quercetin, kaempferol, isorhamnetin and derivatives, soya-saponin glycosides and prodelphinidin type tannins. Moreover, the flowers showed the highest phytochemical content, almost double compared to the leaves [7,8,9]. Recently, it was reported that an extract from *Sulla coronaria* inhibited collagenase and elastase activity in in vitro enzyme assays and increased collagen synthesis in human dermal fibroblasts, indicating its potential use in cosmeceutical treatments for skin problems, including inflammatory, photo degenerative, and aging processes [10]. The key cellular factor in skin senescence is the high production of reactive oxygen species (ROS) that is related to intrinsic elements such as the no more efficient cellular oxidative metabolism and reduced levels of enzymatic and non-enzymatic antioxidants and/or extrinsic environmental factors, such as cigarette smoking, ultraviolet radiation, pollutions [11]. A condition of prolonged oxidative stress (OS) triggers oxidative damage and causes photoaging and inflammatory skin disorders [12]. Many studies have shown that secondary metabolites from plants are valid therapeutic agents for treating OS and inflammation in skin cells [13]; for this reason and to better contribute to a possible cosmeceutical application, in the present study, we evaluated the phytochemical profile by HPLC-DAD, Folin-Ciocalteu, Aluminum Chloride methods to characterize the extract qualitatively and quantitatively. In addition, for the first time, to verify the hypothesis that the extract may be useful to prevent and/or counteract skin aging, the antioxidant and anti-inflammatory activities of *Sulla coronaria* flower’s hydroalcoholic extract were tested on a human dermal fibroblasts (HDFs) cell line.

## 2. Results

### 2.1. Phytochemical Analysis and Antioxidant Feature

With a yield of 4.5% *Sulla coronaria* flower hydroalcoholic extract (SCF), according to Table 1, showed a total phenolic content (TPC) and a total flavonoid content (TFC) of 69.8 ± 0.6 mg GAE/g extract and 15.07± 0.3 mg CE/g extract, respectively (Table 1).

**Figure 2:**
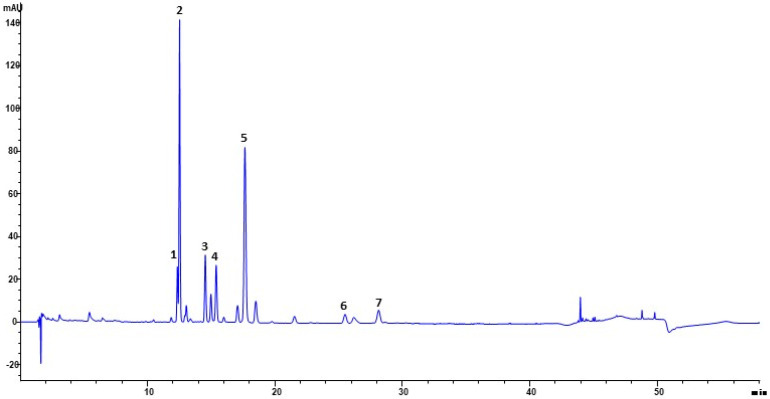
HPLC-DAD phytochemical fingerprint of *Sulla coronaria* flower hydroalcoholic extract. Column: Ascentis Express C18, 15 cm × 4.6 mm, 2.7 µm d.p. The numbers indicating peaks refer to the identified compounds reported in Table 2.

**Figure 3:**
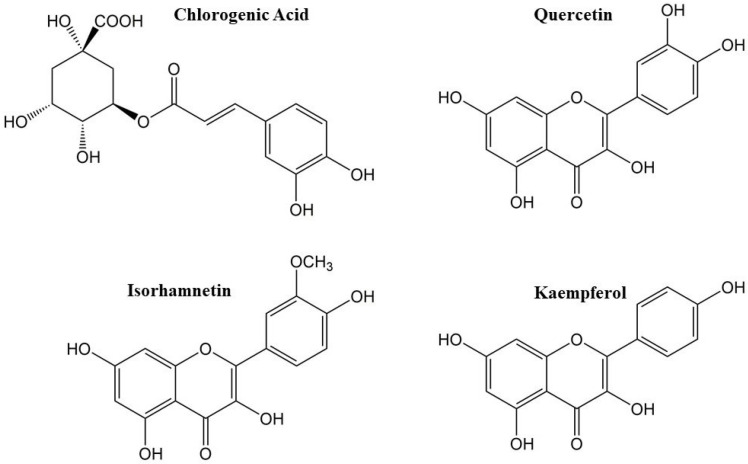
Aglycone chemical structures of the identified compounds in *Sulla coronaria* flower hydroalcoholic extract.

The phytochemical profile of SCF was further investigated by HPLC-DAD analysis, and six compounds were identified from the trace recorded at 330 nm (Figure 2), which had two principal constituents (peaks 2 and 5) along with various smaller peaks. Peak 1 was determined to be chlorogenic acid; peaks 2 and 3 were determined to be a quercetin-derivative and quercetin-3-O-rutinoside respectively; peak 4 was determined to be Isorhamnetin-3-neohesperidoside; and peak 5 was determined to be Quercetin-3-O-glucoside; peak 6 Kaempferol-3-O-rutinoside and peak 7 Isorhamnetin-3-rutinoside (Figure 2 and Figure 3, Table 2). The majority of the remaining unidentified peaks can be attributed to other polyphenolic derivatives. Lastly, SCF was analyzed to establish its antioxidant feature by DPPH assay, resulting in IC_50_ 8.04 ± 0.5 µg/mL (Table 1).

### 2.2. Assessment of Sulla coronaria Flower Extract’s Cytotoxic Potential on HDFs Cell Line

**Figure 4:**
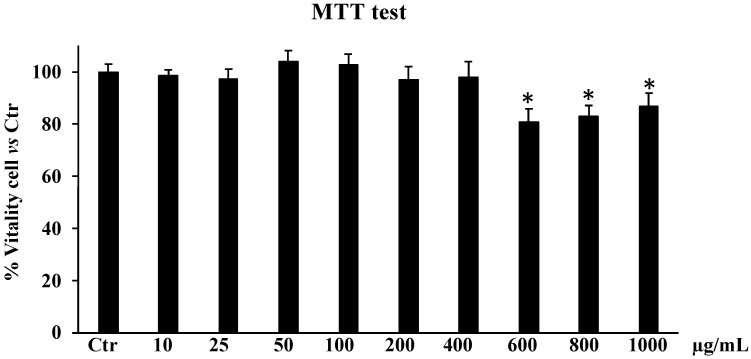
Cytotoxic effect of *Sulla coronaria* flower hydroalcoholic extract on HDF cells. An MTT test was performed on HDFs treated with different concentrations of extract (from 10 to 1000 µg/mL) for 24 h. Data are represented as the means ± S.D. of three independent experiments. Confidence intervals calculated by one-way ANOVA test: * Significant vs. untreated control cells.

First, we assessed the cytotoxic effects of nine different extract concentrations, ranging from 10 to 1000 µg/mL, on HDFs to select the optimal concentration of SCF to employ in our in vitro investigation. A cell viability analysis was then carried out 24 h after the SCF treatment (Figure 4). The data shown in Figure 4 demonstrate that, at all concentrations utilized ranging from 10 to 400 µg/mL, SCF was not cytotoxic to HDF cells.

Starting from 600 to 1000 µg/mL a slight decrease (about 15%) in cell viability was recorded. Due to this modest cytotoxic effect at the concentration of 600 µg/mL, we decided to use only the concentrations of 50, 100, and 200 µg/mL for subsequent experiments, as reported in a previous study [10].

### 2.3. Sulla coronaria Flower Extract Dispalays Anti-Inflammatory Properties

#### 2.3.1. *Sulla coronaria* Flower Extract Reduced Reactive Oxygen Species Levels in IL-1β-Stimulated HDF Cells

**Figure 5:**
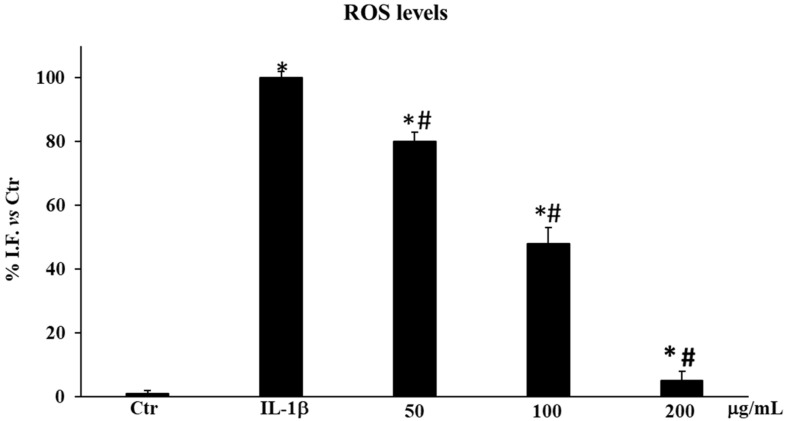
ROS production in HDF untreated cells (Ctr), treated for 12 h with IL-1β (10 ng/mL), and pre-treated for 24 h with the extract (50–100–200 μg/mL). Results are expressed as the percentage of the intensity of fluorescence (I.F.) *vs* Ctr. Values are the mean ± S.D. of three experiments in triplicate. Confidence intervals calculated by one-way ANOVA test: * Significant vs. untreated control cells: *p* < 0.05; # Significant vs. IL-1β-Stimulated cells: *p* < 0.05.

To assess the antioxidant effect of SCF, we examined ROS generation in HDF cells exposed to the extract at the chosen doses (50, 100, and 200 µg/mL). Figure 5 illustrates the extract influence on HDFs’ generation of ROS. In fact, in the pretreatment, our data demonstrated that ROS levels were significantly reduced at all used concentrations compared to IL-1β-stimulated control cells (# *p* < 0.05). In particular, the percentage values of ROS levels were 80.18 ± 0.05% (50 µg/mL), 48.09 ± 0.05% (100 µg/mL), and 5.12 ± 0.1% (200 µg/mL) compared with 100% of the IL-1β-stimulated control cells (Figure 5).

#### 2.3.2. *Sulla coronaria* Flower Extract Inhibited NO• Production in IL-1β-Stimulated HDF Cells

**Figure 6:**
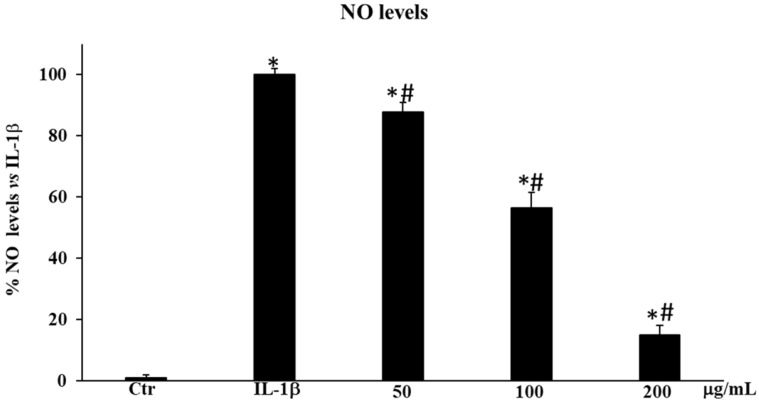
Effect of *Sulla coronaria* flower extract on NO• production in HDF cells. A Griess assay was performed on the supernatant of IL-1β-stimulated cells non-treated and treated with the extract (50, 100, and 200 µg/mL) for 24 h. Data are represented as the means ± SD of three independent experiments. Confidence intervals calculated by one-way ANOVA test: * Significant vs. untreated control cells: *p* < 0.05; # Significant vs. IL-1β-Stimulated cells: *p* < 0.05.

To assess the anti-inflammatory properties of SCF, we measured nitric oxide (NO•) levels in HDF cells non-treated and pre-treated with the three selected concentrations of extract (50, 100, and 200 µg/mL) after 12 h of exposition to IL-1β. Our data showed that the pretreatment with the extract was able to significantly decrease the NO• level compared to HDF cells only treated with IL-1β (* *p* < 0.05) in a dose-dependent manner, reaching about 85% of inhibition at 200 µg/mL (Figure 6).

## 3. Discussion

Naturally occurring plants are invaluable allies in the battle against climate change because of their innate resilience and adaptability. *S coronaria* can be extremely helpful in reducing the negative effects of climate change because of its innate ability to withstand severe environments [14]. Because of their anti-inflammatory, antioxidant, and antibacterial activities, plant extracts are becoming increasingly popular for their curative properties in treating skin-related problems [15]. Moreover, they present a wide range of benefits, including anti-aging properties, as they protect skin from environmental damage [12]. Recently, it was suggested the potential use of *Sulla coronaria* extracts in cosmetic treatments for skin disorders [10].

In this study, the results of the chemical characterization indicated that SCF had a notable concentration of polyphenols (Table 1), and according to previous studies [7,11], the qualitative HPLC analysis of the extract showed the presence of chlorogenic acid and flavonoid compounds, in particular flavonols glycosylates derivatives of quercetin, isorhamnetin, and kaempferol (Figure 2 and Figure 3, Table 2). The chemical characteristics and biological activities of flavonols are greatly influenced by the hydroxyl groups connected to their structure, which can vary in number and position. Due to the high presence of free hydroxyl groups, flavonols show powerful antioxidant power, with quercetin being one of the most potent [16,17]. These results regarding the composition of SCF extract suggested that its phytochemical components could exert significant antioxidant action. The supposed good antioxidant properties of SCF were confirmed by the DPPH test, which showed a significative value of IC_50_ of just 8.04 ± 0.5 µg/mL (Table 1) and is in agreement with the significative fraction of flavonols and the good amount of polyphenols. Burlando et al. reported marked differences in total phenolic content between extracts sampled at two distant sites in the north of the geographic range of the species, specifically in central Italy, and in western Italy, the latter being close to the boundary of the species range [10]. The flowers of wild *Sulla coronaria* used in this study were collected in Corleone, Sicily, in the full center of the native area [18], and the extract obtained showed the highest TPC, TFC, and DPPH values among the three extracts. Although it is necessary to point out the different experimental conditions, these results highlight that a plant’s place of origin may affect quantitatively the phytocomplex of a species.

As a part of the connective tissue, HDFs play a vital role in skin maintenance, repair, and regeneration through the formation and protection of the extracellular matrix, which includes collagen and elastin [19]. They are, nevertheless, also vulnerable to OS, or rather the imbalance between the cell skin’s capacity to eliminate ROS generation with endogenous antioxidants. In dermal fibroblasts, environmental stressors such as pollution, UV radiation, and smoking can raise ROS levels dramatically, which can cause cellular damage, premature aging, and reduced skin function [12]. Given the reported significant induction of collagen production on HDFs and the inhibitory activity of collagenase and elastase by in vitro enzyme assays [10], we decided to investigate the antioxidant and anti-inflammatory potential activities of SCF in HDFs stimulated by IL-1β, a pro-inflammatory cytokine.

Our results clearly demonstrated that the marked ROS production induced by IL-1β exposure in HDFs was significantly counteracted by the pretreatment with SCF extract, particularly at the concentration of 200 µg/mL (Figure 5). The antioxidant effects can be due to the different components of the phytocomplex, such as the flavonols [15]. In fact, it has been reported that, in aged HDFs, quercetin directly decreased the levels of ROS either extracellularly or intracellularly [20]. Furthermore, isorhamnetin was reported to contrast cellular ROS production induced by ultraviolet radiation, preventing UVB-induced cell damage and death in human keratinocytes [21], and the scavenging effect of intracellular ROS by kaempferol was demonstrated during the inflammation and cell death caused by 12-O-tetradecanoylphorbol-13-acetate in HDFs [22]. Additionally, several studies have shown that kaempferol and quercetin can reduce signs of oxidative stress-induced skin aging, including loss of skin elasticity, wrinkle formation, and inflammatory reactions [22,23]. Here, the reported results fit well with these reports, supporting the correlation between the phytochemical composition of the extract and antioxidant/cytoprotective effects.

It is known that the pathophysiology of many diseases, including skin disorders, is significantly influenced by the interdependent processes of OS and inflammation [24]. NO• is involved in many physiological and pathological processes, including inflammation [25]. Tumor necrosis factor-alpha (TNF-α) and IL-1β, two inflammatory cytokines, increase the expression of inducible nitric oxide synthase (iNOS) in cells, which increases NO• production. Elevated NO• levels activate the NF-κB and MAPK signaling pathways, responsible for the inflammatory response progression [25]. Prolonged and excessive NO• generation by iNOS is a contributing factor to tissue damage and sustained OS in chronic inflammatory diseases.

In the present study, following the same trend registered for the ROS levels, SCF extract was substantially reduced in a dose-dependent manner, the NO• amount in HDFs activated by IL-1β (Figure 6), suggesting a protective effect exerted by the extract pretreatment on the inflammation process sustained by a condition of OS. These data are in agreement with previous studies which reported that quercetin and isorhamnetin, and the caffeoylquinic acid derivative, chlorogenic acid, can inhibit the expression of iNOS both in vitro and in vivo with different mechanisms of action [21,26,27]. They can also directly scavenge reactive nitrogen species such as peroxynitrite, a highly reactive oxidant produced when superoxide (O_2_•−) and NO• react, responsible for tissue damage induced by prolonged inflammation.

The present in vitro study demonstrated that the hydroalcoholic extract from *Sulla coronaria* flowers preserves the cellular redox state and counteracts the inflammation response on HDFs induced by IL-1β exposure, thus contributing to the prevention of skin damage and skin aging.

## 4. Materials and Methods

### 4.1. Chemicals and Reagents

Analytic-grade organic solvents, dimethyl sulfoxide (DMSO), 5,5-ditiobis-2-nitrobenzoic acid (DTNB), 2′,7′-dichlorofluorescein diacetate (DCFH-DA), and 2,2-diphenyl-1-picrylhydrazyl (DPPH) were acquired from VWR (Milan, Italy). Unless otherwise stated, all additional substances were obtained from Sigma-Aldrich (Milan, Italy).

Unless otherwise stated, all the supplies and media used in cell culture were purchased from ThermoFisher Scientific in Milan, Italy.

### 4.2. Plant Material and Extraction Procedure

The flowers of wild *Sulla coronaria* were collected randomly at Bona Furtuna organic farm in Corleone (Palermo, Sicily, Italy) (37.77054, 13.29654) at the end of June 2023. The specimen was authenticated by one of the authors (P.M.), and a voucher specimen of the plant (No. 06/23) was deposited in the herbarium of the Department of Drug and Health Sciences, Section of Biochemistry. Fresh flowers were stored at minus 80 °C until used. Subsequently, a crushed portion of 100 g was extracted at 40 °C in 50% ethanol (1:5), with continuous stirring for one hour, three times. The hydroalcoholic extract solution was then filtered and evaporated under reduced pressure with a rotatory evaporator, obtaining an aqueous extract concentrated at 20:1. The aqueous crude solution was then lyophilized in a Freeze Dryer (Heto PowerDry LL3000, Thermo Fisher Scientific Inc., Waltham, MA, USA), and the obtained dried material (4.75 g) was used for the subsequent tests.

### 4.3. Determination of the Total Phenols and Flavonoids Content

The total phenols content (TPC) and the total flavonoid content (TFC) of the leaf extract were measured spectrophotometrically [28]. In particular, TPC, determined by the Folin–Ciocalteau method, was compared to a calibration curve of a known amount of gallic acid and expressed as mg of gallic acid equivalent (GAE/g extract). TFC, analyzed by aluminum chloride methods, was compared to a calibration curve of a known amount of catechin and expressed as mg of catechin equivalent (CE/g extract). The data were obtained from three independent determinations.

### 4.4. Determination of Antioxidant Activity by DPPH Test

Free radical-scavenging capacity was measured with DPPH test that determines spectrophotometrically the ability of the total extract to bleach the stable radical DPPH. The results were compared to Trolox (IC_50_: 15 µM), a water-soluble derivative of vitamin E used as a reference compound. Briefly, after 10 min at room temperature, the absorbance at λ = 517 nm of the DPPH reaction mixture containing different concentrations of *Sulla coronaria* (1–15 µg/mL) in 1 mL of ethanol was recorded [12]. Results, expressed as a decrease in absorbance, represent the average + S.D. of three independent experiments.

### 4.5. HPLC-DAD Analysis

The extract’s polyphenolic fingerprinting was assessed using high-pressure liquid chromatography. A Shimadzu LC 20 (Kyoto, Japan) equipped with a 150 mm × 4.6 mm i.d., 2.7 µm Ascentis Express C 18 column, and a diode array detector (DAD) was used for HPLC-DAD studies. H_2_O/H_3_PO_4_ (99:1, solvent A) and MeOH/CAN/H_3_PO_4_ (49, 5:49, 5:1, solvent B) were the mobile phases. The gradient that was employed was as follows: 95% of solvent A concentration increased to 77% (34 min), remaining at 77% (3 min), 74% (60 min), 60% (85 min), 20% (90 min), and 0% (92 min). A total of 105 min passed. A constant temperature of 25 °C was maintained in the column. The injection volume was 5 µL, and the flow rate was 1 mL/min. The chromatogram profiles were observed at 280 and 330 nm ± 2 nm and recorded between 190 and 500 nm. All the reference compounds were obtained from PhytoLab GmbH & Co., Ltd. (Vestenbergsgreuth, Germany) [29].

### 4.6. Cell Culture and Treatments

The ATCC^®^ Human Dermal Fibroblasts cell line (PCS-201-012, Rockville, MD, USA) was cultivated in DMEM supplemented with 4.5 g/L glucose, 100 U/mL penicillin, and 100 µg/mL streptomycin alongside 15% *v/v* fetal bovine serum (FBS). Cells were treated at subconfluent conditions and cultured at 37 °C in a humidified environment with 5% CO_2_ to ensure similar experimental conditions for all the experiments. Treatments with *Sulla coronaria* were performed by adding different concentrations of extract (50, 100, and 200 µg/mL) to the culture medium for 24 h and after inducing inflammation in cells with IL-1β (10 ng/mL) (recombinant human Il-1β, PeproTech EC, London, UK) for 12 h.

### 4.7. Cell Viability by MTTassay

Various quantities of extract (from 10 to 1000 µg/mL) were applied to HDF cells over a duration of 24 h. Since it measures how tetrazolium salts are converted to colored formazan in the presence of metabolic activity, the MTT test assesses cell viability. The quantity of live cells is directly correlated with the amount of formazan. Using a microplate spectrophotometer reader (Titertek Multiskan, Flow Laboratories, Helsinki, Finland) set at λ = 570 nm, the absorbance of the converted formazan was determined. The outcomes are displayed as a percentage of cell viability relative to 100% of untreated control cells.

### 4.8. Reactive Oxygen Species Assay

2′,7′-Dichlodihydrorofluorescein diacetate (DCFH-DA) was used as a fluorescent probe to measure the amount of ROS [30]. The fluorescence, which is associated with the oxidized radical species 2′,7′-dichlorofluorescein, DCF, was seen using spectrofluorimetry (excitation wavelength: 488 nm; emission wavelength: 525 nm). The overall protein content of each sample was ascertained, and the outcomes were then evaluated as a percentage of fluorescence intensity/mg protein compared to the control. The protein concentration was determined by measuring the absorbance difference between wavelengths of 280 and 260 nm using a microplate reader Sinergy HT, Bioteck, Milan, Italy.

### 4.9. Measurement of NO• Release

The inhibitory effect of the total extract on NO• production was defined by measuring nitrite levels with Griess reagent [29]. HDF cells were pre-treated with different concentrations of *Sulla coronaria* flower’s hydroalcoholic extract (50, 100, and 200 µg/mL) for 24 h and successively stimulated with IL-1β (10 µg/mL) for 12 h. At the end of the treatments, the culture medium (250 µL) was mixed with 250 µL of Griess reagent and incubated at room temperature for 10 min according to manufacturer instructions. The assay is based on the reaction of diazocopulation of nitrite with the Griess reagent. The nitrite content in culture media was determined at λ = 540 nm using a Synergy HT plate reader (BioTek Instruments, Inc., Winooski, VT, USA). Results were calculated by comparison with OD540 of standard solutions of sodium nitrite prepared in H_2_O and expressed as a percentage of nitrite production with respect to IL-1β stimulated untreated cells.

### 4.10. Statistical Analysis

One-way analysis of variance (ANOVA), followed by Bonferroni’s *t*-test, was performed to estimate significant differences among groups. Data were reported as mean values ± S.D. of three experiments in triplicate, and differences between groups were considered to be significant at *p* < 0.005.

## 5. Conclusions

Valorizing naturally occurring plants, often considered weeds, is one exciting field of research. These plants are invaluable allies in the battle against climate change because of their innate resilience and adaptability. Valorization entails identifying and maximizing these resilient plants’ ecological, nutritional, medicinal, and economic advantages. The rising demand for natural and organic cosmetics among consumers stimulates the research of innovative plant-based ingredients for the industry of well-being. Because of their effectiveness and safety profile, they are becoming progressively popular in dermatology and cosmetics. Data showed in this study pointed out that the rich and valuable phytocomplex contained in SCF with its bioactive molecules mostly represented by quercetin, isorhamnetin, and kaempferol derivatives can preserve HDFs from the harmful effects of IL-1β, counteracting the gain in ROS and NO• levels raised by cytokine exposure.

The scavenging effect of these phytochemicals is well documented and demonstrated by the good antioxidant values highlighted for the crude extract by the DPPH test. The ability of SCF to shield from harmful cellular ROS production and the interdependent inflammatory processes by lowering NO levels protects HDFs from cell death and damage, thus decreasing the risk of wrinkles appearance, inflammatory reactions, and other signs of oxidative stress-induced skin aging.

These results and other data from the literature and its safety show that *Sulla coronaria* flowers are suitable for developing natural ingredients for skincare products with antioxidant and anti-inflammatory properties.

## Figures and Tables

**Table 1:** Spectrophotometric quantitative determination of total polyphenols and flavonoids and DPPH test in *Sulla coronaria* flower hydroalcoholic extract (SCF).

	Total Polyphenols (mg GAE/g extract)	Total Flavonoids (mg CE/g extract)	DPPH Test IC_50_ (μg/mL)
SCF	69.8 ± 0.6	15.07 ± 0.3	8.04 ± 0.5 µg/mL
Trolox			15 µM ± 0.62

**Table 2:** Phenolic compounds identified in *Sulla coronaria* flower hydroalcoholic extract by HPLC-DAD.

Peack	Compound	Wavelength nm λ	Ret. Time (min)
1	Chlorogenic acid	330	12.34
2	Quercetin derivative	330	12.51
3	Quercetin-3-O-rutinoside	330	14.54
4	Isorhamnetin-3-neohesperidoside	330	15.40
5	Quercetin-3-O-glucoside	330	17.66
6	Kaempferol-3-O-rutinoside	330	25.46
7	Isorhamnetin-3-rutinoside	330	28.11

## Data Availability

Data were generated at the Department of Drug and Health Science, University of Catania. Data supporting the results of this study are available from the corresponding authors on request.
